# Empirically derived phenotypic subgroups – qualitative and quantitative trait analyses

**DOI:** 10.1186/1471-2156-4-S1-S15

**Published:** 2003-12-31

**Authors:** Marsha A Wilcox, Diego F Wyszynski, Carolien I Panhuysen, Qianli Ma, Agustin Yip, John Farrell, Lindsay A Farrer

**Affiliations:** 1 Genetics Program, Department of Medicine, Boston University School of Medicine, 715 Albany Street, Boston, Massachusetts, 02118 USA

## Abstract

**Background:**

The Framingham Heart Study has contributed a great deal to advances in medicine. Most of the phenotypes investigated have been univariate traits (quantitative or qualitative). The aims of this study are to derive multivariate traits by identifying homogeneous groups of people and assigning both qualitative and quantitative trait scores; to assess the heritability of the derived traits; and to conduct both qualitative and quantitative linkage analysis on one of the heritable traits.

**Methods:**

Multiple correspondence analysis, a nonparametric analogue of principal components analysis, was used for data reduction. Two-stage clustering, using both k-means and agglomerative hierarchical clustering, was used to cluster individuals based upon axes (factor) scores obtained from the data reduction. Probability of cluster membership was calculated using binary logistic regression. Heritability was calculated using SOLAR, which was also used for the quantitative trait analysis. GENEHUNTER-PLUS was used for the qualitative trait analysis.

**Results:**

We found four phenotypically distinct groups. Membership in the smallest group was heritable (38%, p < 1 × 10^-6^) and had characteristics consistent with atherogenic dyslipidemia. We found both qualitative and quantitative LOD scores above 3 on chromosomes 11 and 14 (11q13, 14q23, 14q31). There were two Kong & Cox LOD scores above 1.0 on chromosome 6 (6p21) and chromosome 11 (11q23).

**Conclusion:**

This approach may be useful for the identification of genetic heterogeneity in complex phenotypes by clarifying the phenotype definition prior to linkage analysis. Some of our findings are in regions linked to elements of atherogenic dyslipidemia and related diagnoses, some may be novel, or may be false positives.

## Background

Contemporary advances in medicine are due, at least in part, to the long history of research conducted in Framingham. To date, the majority of the outcome measures have been univariate qualitative or quantitative traits. The objectives of the present analyses were to derive multivariate qualitative and quantitative traits empirically, to examine the heritability of the traits, and to conduct genome-wide linkage analyses with a trait that demonstrated some heritability. The analyses were conducted in the families collected by the Framingham Heart Study made available to participants in the Genetic Analysis Workshop 13.

## Methods

### Population

This study was conducted in the sample from the Framingham Heart Study distributed to participants in Genetic Analysis Workshop 13. The most extreme measurement category across all of the measures for an individual was used to create the multivariate phenotypes. For example, if, over the course of the available measurements, the maximum triglyceride level reached the fourth quartile, the summary measure was the fourth quartile. Continuous measures were categorized according to classes commonly used in clinical practice as follows: body-mass index (BMI) (underweight, normal weight, overweight, obese); tobacco use (none, less than one pack per day, one to two packs per day, two to three packs per day, and more than three packs per day); alcohol use (abstinence, moderate use, heavy use); systolic blood pressure (sbp) [low (< 80), normal (80–129), elevated (130–139), high (>140)]; cholesterol (normal, borderline, high); glucose (low, normal, impaired, hyperglycemic); atherogenic dyslipidemia [no criteria, either lowest HDL quartile, or highest triglyceride decile, both low HDL and high triglycerides (atherogenic dyslipidemia)]. High density lipoproteins (HDL) and triglycerides were characterized in age- and gender-specific quartiles as observed in the Framingham Heart Study data. An individual was classified as having high blood pressure if they were being treated for hypertension, regardless of the clinical measurement.

### Statistical methods

The strategy for the development of qualitative and quantitative traits included nonparametric data reduction, iterative two-staged clustering on the observed dimensions, and the assignment of probability of cluster membership in each cluster for each individual.

Principal components analysis (PCA) is a method commonly used for data reduction. PCA is based upon a Pearson product-moment correlation which assumes a pair-wise Gaussian structure. The original continuous data were not pair-wise normal and did not meet the assumptions for this method. Multiple correspondence analysis (MCA) is a nonparametric data reduction method free of the assumptions underlying PCA. The only requirement for MCA is a non-negative rectangular data matrix. MCA uses a singular value decomposition (SVD) of the matrix. Eigenvalue (vector) decomposition is a special case of SVD. The objective of MCA is to identify a low-dimensional subspace that comes closest to all of the data points. It is analogous to graphing the results of a factor analysis in a multidimensional Euclidean space. However, the space identified in MCA is not Euclidian. The coordinates of each individual in the identified multi-dimensional space served as the basis for the identification of subgroups or clusters [1].

**Figure 1:**
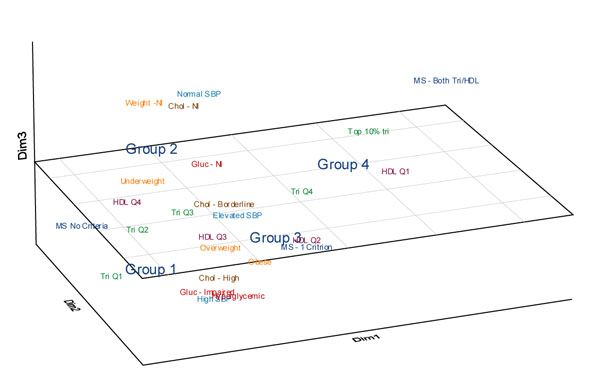
Simple Correspondence Analysis – Relationships Among Phenotypic Subgroups

Each study participant with phenotype data was assigned a score on each of the eight retained dimensions (data not shown). Next, a multistaged clustering strategy was used to identify distinct subgroups [2]. It is not unusual for groups identified with clustering techniques to be subject to the idiosyncrasies of the estimation data set. In an attempt to mitigate that difficulty, we first conducted repeated k-means clustering with different random cluster seeds and used a larger k (number of clusters) than we expected in the data. Groups that consistently clustered together across all of the initial analyses were identified as intact clusters. An agglomerative hierarchical clustering algorithm was then implemented using the intact clusters and the remaining individuals in the sample. A comparison of the within- to between-group variation on items used to form the groups and group profiles on other variables provided the basis for the selection of the final subgroup structure. Simple correspondence analysis was used to create a graphical representation of the relationships of the subgroups with each other and the categories used to identify the groups (see Figure 1). The "corem", "defac", "recip/semis", and "parti/decal" modules of SPAD software [3] were used for both the multiple correspondence analysis and the clustering algorithms. SAS software [4] was used for the simple correspondence analysis. S-PLUS [5] was used to produce the graph.

It is possible to represent cluster membership as both qualitative and quantitative traits. The qualitative trait is membership in the cluster, which is binary. The quantitative trait represents the degree of affiliation with the cluster, distance from the cluster centroid, or probability of membership. To compare the utility of the measures and the consistency of the linkage results, both traits were constructed and linkage analyses were conducted on each.

Binary logistic regression was used to estimate the probability of cluster membership for each study participant in each of the clusters. The natural logarithm of the probability of membership in Group 4 (described below) was the dependent measure in the quantitative trait analyses. Categorical cluster membership was used in the qualitative trait analyses. Two-point variance components linkage analysis was conducted using SOLAR [6]. Multipoint NPL (nonparametric linkage) analysis was performed using the S (pairs) option of GENEHUNTER-PLUS, and maximizing nonparametric LOD scores ("K&C LOD scores") were calculated under an exponential model with δ constrained between 0 and 2 [7].

## Results

### Correspondence analysis and clustering

Coordinates on eight axes (analogous to factor scores) were retained and used for clustering. Four clusters were identified. The cluster sizes were *n *= 1030 (35.7%), *n *= 670 (23.22%), *n *= 881 (30.54%), and *n *= 304 (10.54%).

An index measure of the prevalence of each of the independent variables within each of the clusters was calculated by dividing the observed category proportion in a cluster by its expectation, the marginal proportion. If the prevalence in a cluster did not differ from the sample, the index would be unity. Group 1 had indices higher than 1.25 for the first quartile triglyceride measure (2.02), high blood pressure (1.53), high cholesterol (1.44), hyperglycemia (1.43), fourth quartile HDL (1.39), and heavy alcohol use (1.27). Group 2 was characterized by high HDL (1.62) and lower rates of all other measures. This was a particularly healthy group. Group 3 was characterized by low HDL, obesity, and high triglycerides (1.65, 1.31, and 1.24, respectively). The last group (Group 4) contained all of the individuals in the sample who met the criteria for atherogenic dyslipidemia as defined by lowest quartile for HDL and highest decile for triglycerides. They had high indices for atherogenic dyslipidemia (8.39), top decile for triglycerides (5.24), lowest quartile HDL (3.82), obesity (1.51), and smoking (1.49).

Figure 1 shows a simple correspondence analysis graph of the relationships between the groups and each of the categories used to identify them. The four-group structure is fully represented in the three-dimensional display. Group 4 is nearest the criteria for atherogenic dyslipidemia labeled "MS" on the graph. The measures of good health cluster around Group 2. Groups 1 and 3 have moderate to high levels of most of the independent variables.

### Heritability

The heritability of the probability of group membership was computed using SOLAR. The heritability of each of the quantitative traits is 20% (*p *< 1 × 10^-6^) for Group 1, 19% (*p *< 1 × 10^-6^) for Group 2, 39% (*p *< 1 × 10^-6^) for Group 3, and 38% (*p *< 1 × 10^-6^) for Group 4. Linkage analysis was conducted for the probability of membership in Group 4 and for a binary qualitative trait representing membership in the Group 4.

### Linkage

#### Quantitative trait analysis

Table 1 shows the quantitative trait linkage results. There were three LOD scores above 3, one on chromosome 11 (11q23) and two on chromosome 14 (14q23, 14q31). There were 25 other LOD scores above 2 for this trait at 1p32, 2p23, 3pter, 4q13, 4q21 (*n *= 3), 4q22, 4q28, 5q34, 6p12, 6p21, 8p16, 8q24 (*n *= 2), 9q34, 10q26, 11q13, 15q21, 17p11, 17q24, 18q22 (*n *= 2), 19p13, 22p11.

#### Qualitative trait analysis

In the multipoint NPL analysis, there were two K&C LOD scores above 1.0 (Table 2), one on chromosome 6 (6p21) and another on chromosome 11 (11q23). A LOD score of exactly 1 was observed on chromosome 16 (16q21).

## Discussion and Conclusions

We found four empirically derived, phenotypically distinct subgroups. One group was very healthy, two groups had mild to moderately elevated lipid levels, and one group had lipid levels characteristic of atherogenic dyslipidemia. The profile of the latter group resembled atherogenic dyslipidemia and atherogenic dyslipidemia. Grundy [8] identified atherogenic dyslipidemia as a disorder characterized by elevated triglycerides, small LDL particles, and reduced HDL. The multivariate measure of this related trait had significant heritability (38%) and was chosen for examination in linkage analyses. It should be noted that this cluster was identified empirically. It represents factors associated with atherogenic dyslipidemia. This constellation of factors was chosen empirically, not clinically, for further linkage analyses.

Three loci were common across our qualitative and quantitative analyses. One of the three LOD scores above 3 in the quantitative trait was observed on 11q23. The highest NPL score in the qualitative trait analysis was observed in the same region. Similarly, there were consistent findings on 6p21 and 18q22 in both the qualitative and quantitative analyses.

Several of our results are close to those reported by Aouizerat et al. [9] in a genome scan for familial combined hyperlipidemia. Our results on chromosomes 2q and 11q are in the same regions as the two highest LOD scores reported in that study. Additionally, our highest scores on 10q, 15p, 18p, and 22p are in regions close to those reported in those regions in the same study. Comuzzie et al. [10], Comuzzie [11], Hager et al. [12], and Rotimi et al. [13] all previously reported human obesity quantitative trait loci at the same regions in which we found some evidence for linkage on chromosomes 2q and 17q.

Lindsay et al. [14] reported linkage of diabetes in Pima Indians at the same region on chromosome 14 as two of our three LOD scores over 3. Their reported region of linkage on chromosome 6 maps to the same regions in which we report evidence for linkage to our quantitative trait. Arya et al. [15] used a principal components approach to construct three quantitative traits representing insulin-resistance syndrome. They reported linkage on both chromosomes 6q and 7. Our findings on chromosome 6 were on 6p and likely not related to those shown by Arya et al. We did not have a measurable signal on chromosome 7. This lack of replication is likely due to difference in traits. Our empirically derived trait has more in common with atherogenic dyslipidemia than it does with insulin resistance syndrome.

Our results are also close to those reported by Soro et al. [16]. In a study investigating the genetic etiology of low HDL, they showed linkage on chromosomes 8q23 and 16q24. We found some evidence in the quantitative trait analysis at 8q24, and at 16q21 in the qualitative trait analysis.

For the qualitative trait analyses, one location on chromosome 14p was common across this analysis and another done by this group examining a trait for atherogenic dyslipidemia [17]. Both analyses resulted in K&C LOD scores of approximately 0.6. It appears that the empirically derived qualitative trait is similar to atherogenic dyslipidemia, but not identical.

Some of the present linkage findings are in regions linked to elements of atherogenic dyslipidemia and related diagnoses, some may be novel, or may be false positives. It is also possible that the number of LOD scores above 2 in the quantitative trait analysis is due to the clustering of distinct traits with distinct genetic etiologies rather than a single trait with an oligogeneic or polygenetic etiology.

## Figures and Tables

**Figure 2:**
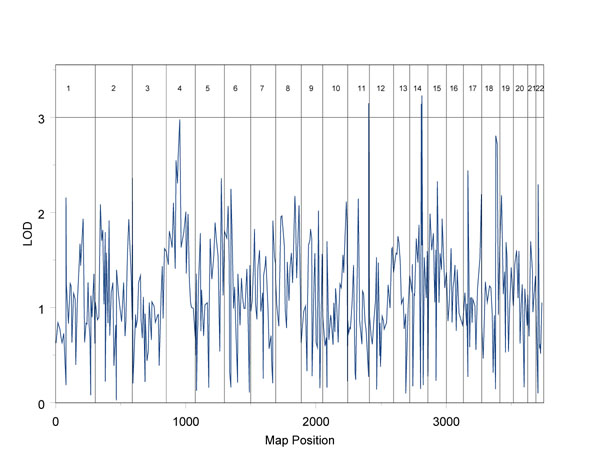
Two-point Linkage Results for Probability of Membership in Group Four (atherogenic dyslipidemia)

**Table 1:** Quantitative Trait Two-Point LOD Score

Chromosome	Marker	Two-Point LOD
1 – 1p32	GATA129H04	2.16
2 – 2p23	GATA11H10	2.09
3 – 3pter	3PTEL25	2.36
4 – 4q22	GATA2F11	2.98
4 – 4q21	ATA2A03	2.61
4 – 4q13	GATA24H01	2.55
4 – 4q21	GATA10G07	2.31
4 – 4q21	GATA7D01	2.10
4 – 4q28	GATA11E09	2.01
5 – 5q34	Mfd154	2.36
6 – 6p12	GGAA15B08	2.25
6 – 6p21	GATA29A01	2.07
8 – 8q16	GATA7G07	2.17
8 – 8q24	UT721	2.08
8 – 8q24	GATA21C12	2.03
9 – 9q34	029xg1	2.02
10 – 10q26	GGAA23C05	2.11
11 – 11q23	109xc3	3.15
11 – 11q13	GATA90D07	2.15
14 – 14q31	GATA193A07	3.23
14 – 14q23	Mfd190	3.14
15 – 15q21	GATA85D02	2.33
17 – 17p11	ATA78D02	2.44
17 – 17q24	217yd10	2.19
18 – 18q22	GATA7E12	2.81
18 – 18q22	ATA82B02	2.72
19 – 19p13	GATA21G05	2.18
22 – 22p11	Mfd313	2.30

**Table 2:** Qualitative Trait – Multipoint NPL Scores, *p*-values, Kong & Cox LOD Scores

Chromosome	Location	NPL (*p*-value)	Kong & Cox LOD

6^A^	41.9 (6p21)	0.612 (0.051)	1.103
10	69.63 (10q11)	0.606 (0.052)	0.760
11^A^	127.86 (11q23)	0.898 (0.009)	1.693
14	22.94 (14p11)	0.607 (0.052)	0.604
16	90.65 (16q21)	0.677 (0.035)	1.000
18^A^	120.7 (18q22)	0.49 (0.094)	0.541

^A^adjacent to QTL LOD score > 2
